# RETRACTED: Jang, S.; Sohn, A. Awareness, Intention to Use Pre-Exposure Prophylaxis, and Factors Associated with Awareness among Men Who Have Sex with Men in the Republic of Korea. *Trop. Med. Infect. Dis.* 2024, *9*, 170

**DOI:** 10.3390/tropicalmed10070178

**Published:** 2025-06-23

**Authors:** Sarang Jang, Aeree Sohn

**Affiliations:** Department of Public Health, Sahmyook University, Seoul 01795, Republic of Korea; srjang@syu.ac.kr

The Journal retracts and removes the article, “Awareness, Intention to Use Pre-Exposure Prophylaxis, and Factors Associated with Awareness among Men Who Have Sex with Men in the Republic of Korea” [1], cited above.

Following publication, concerns were brought to the attention of the publisher regarding the ownership of the data presented in the publication [1].

Adhering to our standard procedure, an investigation was conducted by the Editorial Office and the Editorial Board that confirmed that the data contained in the article are subject to a confidentiality agreement, and the authors did not receive appropriate permission to publish from the Korea Federation for HIV/AIDS Prevention. As retroactive permission could not be obtained, and because of the sensitive and confidential nature of the data set, the Editorial Board have decided to retract and remove the article as per MDPI’s retraction policy (https://www.mdpi.com/ethics#_bookmark30). The title, the authors’ information and this retraction note will remain permanently available, and the article itself has been removed. This information has also been transmitted to the relevant indexing organizations.

This retraction was approved by the Editor-in-Chief of *TropicalMed.*

The authors agree with this retraction.
